# Phylogeography and conservation genetics of the rare and relict *Bretschneidera sinensis* (Akaniaceae)

**DOI:** 10.1371/journal.pone.0189034

**Published:** 2018-01-12

**Authors:** Mei-Na Wang, Lei Duan, Qi Qiao, Zheng-Feng Wang, Elizabeth A. Zimmer, Zhong-Chao Li, Hong-Feng Chen

**Affiliations:** 1 Key Laboratory of Plant Resources Conservation and Sustainable Utilization, South China Botanical Garden, Chinese Academy of Sciences, Guangzhou, China; 2 Shenzhen Key Laboratory for Orchid Conservation and Utilization, The National Orchid Conservation Center of China and The Orchid Conservation & Research Center of Shenzhen, Shenzhen, China; 3 College of Agriculture, Henan University of Science and Technology, Luoyang, China; 4 Department of Botany, National Museum of Natural History, MRC 166, Smithsonian Institution, Washington, D.C., United States of America; National Cheng Kung University, TAIWAN

## Abstract

*Bretschneidera sinensis*, a class-I protected wild plant in China, is a relic of the ancient Tertiary tropical flora endemic to Asia. However, little is known about its genetics and phylogeography. To elucidate the current phylogeographic patterns and infer the historical population dynamics of *B*. *sinensis*, and to make recommendations for its conservation, three non-coding regions of chloroplast DNA (*trn*Q-*rps*16, *rps*8-*rps*11, and *trn*T-*trn*L) were amplified and sequenced across 256 individuals from 23 populations of *B*. *sinensis*, spanning 10 provinces of China. We recognized 13 haplotypes, demonstrating relatively high total haplotype diversity (*h*_T_ = 0.739). Almost all of the variation existed among populations (98.09%, *P* < 0.001), but that within populations was low (1.91%, *P* < 0.001). Strong genetic differentiation was detected among populations (*G*_*ST*_ = 0.855, *P* < 0.001) with limited estimations of seed flow (*N*_*m*_ = 0.09), indicating that populations were strongly isolated from one another. According to SAMOVA analysis, populations of *B*. *sinensis* in China could be divided into five geographic groups: (1) eastern Yunnan to western Guangxi; (2) Guizhou-Hunan-Hubei; (3) central Guangdong; (4) northwestern Guangdong; and (5) the Luoxiao-Nanling-Wuyi -Yangming Mountain. Network analysis showed that the most ancestral haplotypes were located in the first group, i.e., the eastern Yungui Plateau and in eastern Yunnan, which is regarded as a putative glacial refugia for *B*. *sinensis* in China. *B*. *sinensis* may have expanded its range eastward from these refugia and experienced bottleneck or founder effects in southeastern China. Populations in Liping (Guizhou Province), Longsheng (Guangxi Province), Huizhou (Guangdong Province), Chongyi (Jiangxi Province), Dong-an (Hunan Province), Pingbian (Yunnan Province) and Xinning (Hunan Province) are proposed as the priority protection units.

## Introduction

*Bretschneidera sinensis* Hemsl., a relict tree of the Tertiary tropical flora, occurs in evergreen broad-leaved or mixed evergreen and deciduous forests in montane areas at altitudes of 300–1700 m. Historically, *B*. *sinensis* was a heliophilous volunteer species and a long-term resident in the eastern Palearctic [1, 2]. *B*. *sinensis* is listed as a Category-I species in the “Statute of Protection of Wild Plants in China” [3], and is flagged as “NT” (Near Threatened) in the Chinese Species Red List [4], indicating that it requires urgent protection and restoration. Its rare and endangered status is exemplified by its greatly diminished population size in recent decades, probably due to scarcity of flowering individuals, low seed production, weak germination, deforestation, and destructive collection of seedlings [5]. According to Wu’s Angiosperm taxonomic system [2,6], *Bretschneidera sinensis* is endemic to China, mainly distributed in areas south of the Yangtze River, with rare appearances in Vietnam and Laos. Previously, it was generally believed that Bretschneideraceae was an independent monotypic family closely related to Sapindaceae and Hippocastanaceae [7, 8, 9]. However, based on recent morphological and molecular phylogenetic studies [10], the IV Angiosperm Phylogeny Group [11] classified *B*. *sinensis* in the family Akaniaceae, which contains one other monotypic genus endemic to eastern Australia (*Akania*) Hook.f.). Akaniaceae appears most related to Caricaceae, Capparaceae and Moringaceae in Brassicales [11]. As a relict and endemic tree from a monotypic genus, *B*. *sinensis* has attracted the attention of various workers on phylogeny, ancient geography, and climate research [12, 13]. However, little is known about its genetics and phylogeography.

The geographic distribution, demographic history, and patterns of genetic diversification of many plant and animal species are assumed to have been greatly influenced by climatic oscillations during the Pleistocene [14, 15], when at least four major glaciations are thought to have occurred in Asia [16]. Although the glacial advance in Asia was not as extensive as that in Europe and North America, the flora and fauna of Asia are believed to have been affected by the climatic oscillations [17, 18]. Pollen and other fossil records have traditionally provided evidence for inferring biogeographic histories and signatures of glacial refugia [19]. However, in most of the relevant studies on *B*. *sinensis*, either the fossil records were poor or data prior to the Quaternary period were not available [20].

Phylogeography, an interdiscipline of phylogeny and biogeography, disentangles historical changes in patterns of gene flow, isolation, and secondary contact among divergent populations at various spatiotemporal scales [21–23] and helps to identify biodiversity hotspots and to inform conservation policies [14, 15, 24]. It is a powerful tool for inferring the processes that determine the genetic composition of species or species groups [14, 23], and can shed light on the association between climate cycles and species distributions [25].

Owing to their non-recombining nature, moderate evolutionary rates, and generally maternal inheritance, the genomes of angiosperm organelles, particularly those of non-coding regions of chloroplast DNA, have enabled resolution of some phylogeographic questions in angiosperms [14, 18, 19, 22, 26–29]. These regions are sensitive to the effects of habitat fragmentation due to their smaller effective population size relative to nuclear genes, and because of the limits of seed-mediated gene dispersal compared to pollen-mediated gene flow [27, 30].

In the present study, we aimed to clarify the phylogeography of *B*. *sinensis* using three chloroplast DNA markers. Our specific objectives were as follows: (1) to understand the historical processes that have affected the distribution of *B*. *sinensis*; (2) to identify refugia of the species; and (3) to make recommendations for future conservation activities.

## Methods

### Sampling and DNA extraction

**Fig 1:**
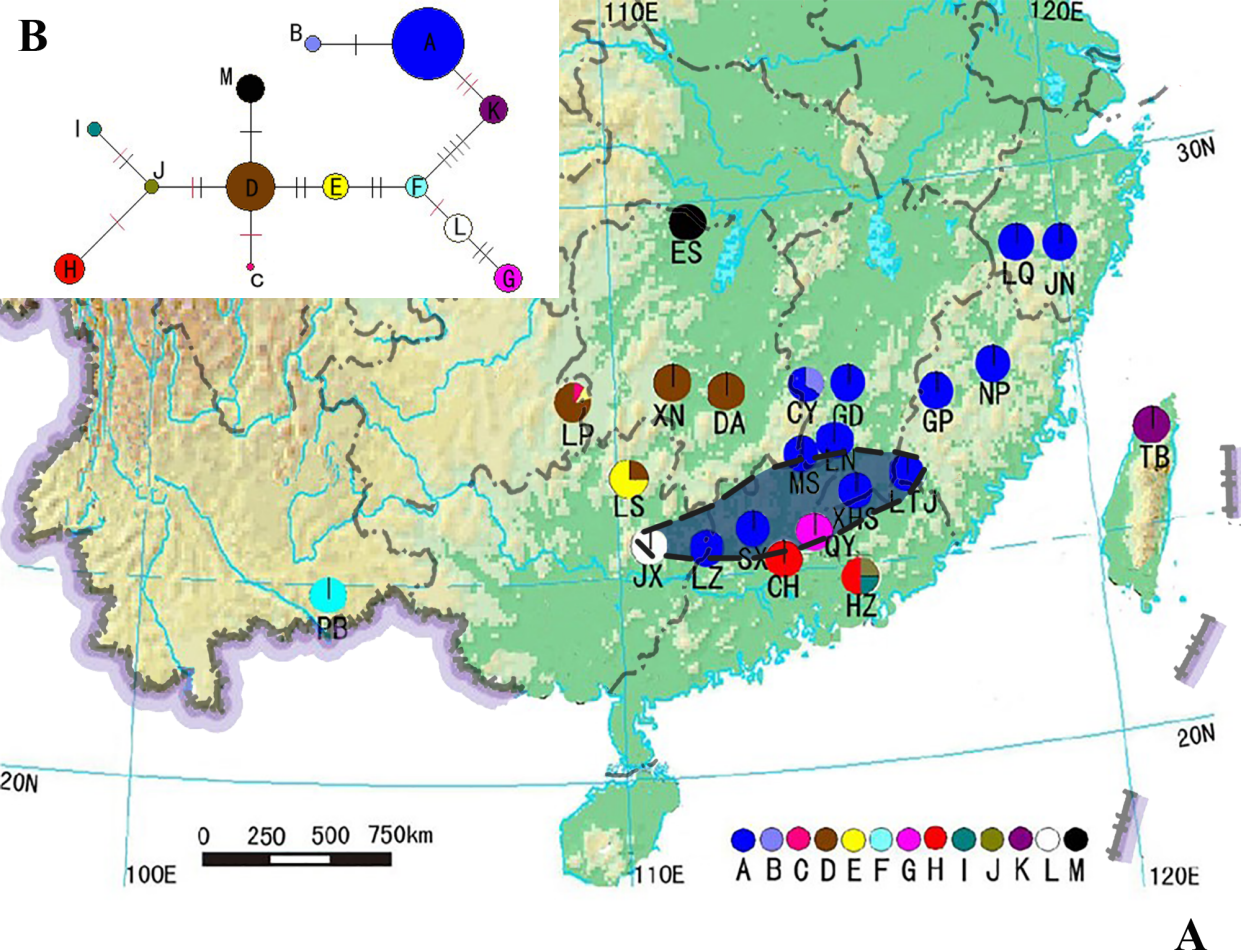
**Distribution (A) and network (B) of *Bretschneidera sinensis* haplotypes.** (A) Distribution of 13 *Bretschneidera sinensis* haplotypes in China. Letters below the colorful circles in the map stand for the population names; different colorful circles are to distinguish the haplotypes (see Legend); the shadow area with dashed margin indicades the approximate coverage of Nanling Mtn. (B) Network of *Bretschneidera sinensis* haplotypes in China. Median-joining network of the 13 haplotypes of *B*. *sinensis*. Solid bars indicate the number of mutation steps, black bars represent parsimony sites, and red bars represent cpSSR variation. The size of circles reflects the frequency of haplotypes observed.

Leaf materials were collected throughout the range of *B*. *sinensis*, from 10 provinces in China. No specific permissions were required by authorities of the collecting areas, on the condition that there be no harm to the target plants. It is not illegal to remove only a few leaves from a protected tree species for the purpose of scientific research. For each population, leaves were collected from individuals that were separated over 30 m. In total, 256 individuals from 23 populations were investigated (Table 1, Fig 1A). Unfortunately, due to habitat loss, only one population (PB, see Fig 1A) was found in Yunnan. However, our collecting area did not cover all the recorded localities of *B*. *sinensis* in this province, e.g. Hengduan Mtn. Another trip is planned for other populations.

**Table 1:** Sampling sites, sample sizes (n), haplotype diversity (*h*), and nucleotide diversity (*π*) of the 23 *Bretschneidera sinensis* populations investigated in this study. Standard errors are given in parentheses.

Population	Sample location	Mountain region	Latitude (°N)	Longitude (°E)	n	Hap- lotype	*h* (sd.)	*π*×10^−3^ (sd.)
1. ES	Enshi, Hubei	Xingdo-u Mtn.	30°2'54"	109°8'1"	12	M	0(0)	0(0)
2. GD	Guidong, Hunan	Bamian Mtn.	25°56'22"	113°40'48"	12	A	0(0)	0(0)
3. DA	Dong-an, Hunan	Shunhu-ang Mtn.	26°23'51"	111°17'59"	12	D	0(0)	0(0)
4. XN	Xinning, Hunan	Ziyun Mtn.	26°36'31"	111°05'26"	12	D	0(0)	0(0)
5. JN	Jingning, Zhejiang	Donggo-ng Mtn.	27°58'24"	119°38'9"	12	A	0(0)	0(0)
6. LQ	Longquan, Zhejiang	Fengyan-g, Mtn.	28°4'29"	119°8'29"	12	A	0(0)	0(0)
7. CY	Chongyi, Jiangxi	Qiyun Mtn.	25°40'55"	114°18'30"	11	A, B	0.5091 (0.1008)	0(0)
8. LN	Longnan, Jiangxi	Jiulian Mtn.	24°54'40"	114°47'23"	12	A	0(0)	0(0)
9. LP	Liping, Guizhou	Taiping Mtn.	26°13'49"	109°8'12"	12	D, E, C	0.3182 (0.1637)	0.074 (0.120)
10. NP	Nanping, Fujian	Wuyi Mtn.	26°38'30"	118°10'39"	12	A	0(0)	0(0)
11. GP	Guanping, Fujian	Wuyi Mtn.	27°47'52"	117°42'34"	12	A	0(0)	0(0)
12. PB	Pingbian, Yunnan	Dawei Mtn.	22°59'1"	103°41'15"	8	F	0(0)	0(0)
13. LS	Longsheng, Guangxi	Huaping	25°37'31"	109°55'11"	12	D, E	0.4091 (0.1333)	0.182 (0.202)
14. JX	Jinxiu, Guangxi	Dayao Mtn.	24°09'35"	110°12'42"	12	L	0(0)	0(0)
15. CH	Conghua, Guangdong	Daling Mtn.	23°32'54"	113°35'11"	9	H	0(0)	0(0)
16. HZ	Huizhou, Guangdong	Nankun Mtn.	23°37'57"	113°52'6"	12	H, I, J	0.6818 (0.0910)	0.181 (0.201)
17. SX	Shixing, Guangdong	Cheba Mtn.	24°42'21"	114°15'35"	12	A	0(0)	0(0)
18. QY	Qingyuan, Guangdong	Dabang Mtn.	24°43'34"	112°17'13"	12	G	0(0)	0(0)
19. LZ	Lianzhou, Guangdong	Dadong Mtn.	24°46'24"	112°41'20"	12	A	0(0)	0(0)
20. LTJ	LongTan Jiao Guangdong	Nanling Mtn.	24°55'28"	113°1'58"	10	A	0(0)	0(0)
21. XHS	XiaoHuang Shan Guangdong	Nanling Mtn.	24°58'43"	113°1'58"	7	A	0(0)	0(0)
22. MS	Mangshan Hunan	Nanling Mtn.	24°58'43"	112°51'3"	7	A	0(0)	0(0)
23. TB	Taibei, Taiwan		25°7'34"	121°51'34"	12	K	0(0)	0(0)

Total genomic DNA was extracted from leaf tissue dried in silica gel using a slightly modified protocol of the CTAB method [31]. DNA was finally dissolved in 100 μL TE-buffer for long-term storage at -20°C.

### PCR amplification and direct sequencing

An initial screen for DNA sequence variability of various chloroplast markers using universal primers was conducted on four individuals from geographically disjunct populations [32–40]. The plastid regions, *trnL-trnT*, *trnQ-rps16*, and *rps8-rps11*, were chosen for the further study because they produced clear single bands via electrophoresis and exhibited the most sequence variation (Table 2). We amplified *trnL-trnT* with the primer pair L (5′-TGCGATGCTCTAACCTCTGAG-3′) and R (5′-CCAATTAAGTCCGTAGCGTCTAC-3′), *trnQ-rps16* region with primer pair ANU_cp009-L (5′-GCTTTCTACCACATCGTTTT-3′) and ANU_cp010-R (5′-GGTTTTGGTCCCGTTACT-3′), as well as *rps8-rps11* region with ANU_cp099-L (5′-GCTTTTGAATCCACAAGTACC-3′) and ANU_cp100-R (5′-AATGACAGATCGAGAAGCTC-3′) primers. Sequencing of *trnQ-rps16* and *rps8-rps11* was carried out in both directions, using primer pair identical to those used for PCR for *trnQ-rps16*, except that the forward primer for *rps*11-*rps*8 was newly designed (5′-CGGGTTGTCTAATATCATATCACT-3′). Sequencing of *trnL-trnT* was conducted in the forward direction only, using the same primers as used for PCR, because we failed to obtain consistently good sequencing results from the backwards direction.

**Table 2:** Haplotypes of *Bretschneidera sinensis* based on the sequences of *rps8-rps11*, *trnQ-rps16*, and *trnT-trnL* cpDNA intergenic spacers.

Haplotype	*rps8-rps11*					*rps16-trnQ*			*trnT-trnL*						
Mutation No.	1	2	3	4	5	6	7	8	9	10	11	12	13	14	15
A	C	G	-	A	C	(A)_9_	A	A	C	G	-	C	-	(AT)_6_	(A)_10_
B	C	G	-	A	C	(A)_9_	A	A	C	G	-	C	▽	(AT)_6_	(A)_10_
C	C	G	Δ	C	C	(A)_10_	T	T	C	G	A	T	-	(AT)_6_	(A)_10_
D	C	G	Δ	C	C	(A)_10_	T	T	C	G	A	T	-	(AT)_8_	(A)_10_
E	C	G	Δ	C	C	(A)_10_	T	T	C	G	-	C	-	(AT)_8_	(A)_10_
F	C	G	Δ	C	C	(A)_10_	T	T	C	G	-	C	-	(AT)_7_	(A)_10_
G	C	A	Δ	C	C	(A)_10_	T	T	A	G	-	C	-	(AT)_5_ T	(A)_9_
H	T	G	Δ	C	C	(A)_10_	T	T	C	G	A	T	-	(AT)_11_	(A)_10_
I	T	G	Δ	C	A	(A)_10_	T	T	C	G	A	T	-	(AT)_9_	(A)_10_
J	T	G	Δ	C	C	(A)_10_	T	T	C	G	A	T	-	(AT)_10_	(A)_10_
K	C	G	-	A	C	(A)_10_	A	A	C	G	-	C	-	(AT)_7_	(A)_10_
L	C	G	Δ	C	C	(A)_10_	T	T	C	G	-	C	-	(AT)_5_ T	(A)_9_
M	C	G	Δ	C	C	(A)_10_	T	T	C	T	A	T	-	(AT)_8_	(A)_10_

Note: Δ: TTCAAC; ▽: ATACTA

To prepare the PCR work solution, 80 ng of template genomic DNA was combined in a volume of 40 μl, with 4 μl 10× PCR buffer, 100 mM of each of the four dNTPs, 10 μM of each primer, and four units of rTaq DNA Polymerase (Takara). The amplifications were performed in a Robocycler Gradient 96 instrument (Sensoquest, Germany), with an initial denaturation step of 4 min at 94°C, followed by 35 cycles (50 s of denaturation at 95°C, 50 s of annealing [the temperatures for *trn*L-*trn*T, *trn*Q-*rps*16, and *rps*8-*rps*11 were 54°C, 50°C, and 57°C, respectively] and 2 min of extension at 72°C), with a final extension step at 72°C for 10 min. The products were visualized on 1.5% TBE-agarose gels stained with ethidium bromide. PCR products with single bright bands were purified prior to sequencing using an Axygen DNA gel extraction kit (Axygen Biosciences, Union City, USA) and were sequenced using an ABI 3730xL automated DNA sequencer (Invitrogen Trading Shanghai Co., Ltd, China).

### Data analysis

The cpDNA sequences were manually edited and aligned using BioEdit version 7.0.9.0 [41]. Since all the markers were found in chloroplast DNA, the alignments of the three cpDNA regions were combined to produce a single data set. Variable sites in the data matrix were double checked against the original chromatogram. The data analyses included the following four aspects:

#### Genetic diversity and genetic structure analysis

Five indices were used to estimate cpDNA diversity: (1) N_a_, the number of haplotypes found in a given population; (2) *h*, haplotype diversity (Nei, 1987); (3) π, nucleotide diversity; (4) *h*_S_, average within-population diversity; and (5) *h*_T_, total genetic diversity. The *h* and π indices were calculated for each population using Arlequin version 3.5 [42]; *h*_S_ and *h*_T_ were estimated using PERMUT 2.0[43] with 2000 permutations.

To identify patterns of genetic variation within and between populations, we performed hierarchical AMOVA for all populations using Arlequin version 3.5 [42]. In addition, hierarchical analyses dividing populations into different geographic groups were performed. We also compared two measurements of genetic differentiation between populations, *G*_ST_ and *N*_ST_, using PERMUT 2.0[43] with 2000 permutations. The pairwise population *N*_ST_ matrix was calculated in DnaSP version 5.00.05 [44] for all populations and separately for the southern and northern populations.

#### Haplotype genealogy reconstruction

We inferred genetic relationship among haplotypes (defined by all variation including cpDNA SSRs sites) with Network 4.5.1.6 (www.fluxus-engineering.com/network_terms.htm) using the median-joining method [45]. The genetic relationships among haplotypes defined by informative sites and indels were only inferred by TCS 1.2 [46]. Moreover, phylogenetic analysis of haplotypes was performed using PAUP 4.0b10 [47] with *Carica papaya* L. as outgroup. A neighbor-joining (NJ) tree was generated for haplotypes defined by all variable sites, and a maximum-parsimony (MP) tree was generated for haplotypes defined by variable sites excluding cpSSRs sites.

#### Population history dynamic analysis

Neutrality tests to estimate Tajima’s D [48] and Fu and Li’s D* and F* statistics [49] were conducted using the program DNA Sequence Polymorphism (DnaSP version 5.00.05;) [44] to test for evidence of population expansion or selection in the cpDNA. If the above values showed significant (P < 0.05) positive or negative values, we could infer that the populations of the species had experienced a bottleneck. Mismatch distribution analysis was performed using DnaSP version 5.00.05) [44] based on pairwise nucleotide differences between any two individuals within a group.

#### Evaluation of priority protection units

We evaluated the priority of conservation units according to genetic diversity and genetic uniqueness. Populations with higher genetic diversity and more unique genetic constitution warrant a higher priority of protection. We classified haplotypes into five categories: ancestral, endemic, rare, derived and common, and assigned a value to each category according to its importance: 7, 5, 5, 3, and 3, respectively. We then performed simulations on the data using the following formula for genetic importance *H* of a given population:

$$H=Ha+Hd+He+Hr+Hc+10\pi_{i}/\pi$$

Where *Ha*, *Hd*, *He*, *Hr*, and *Hc* are the values for the ancestral, derived, endemic, rare, and common haplotypes, respectively; π_*i*_ is the nucleotide diversity for population *i*; and *π* is the nucleotide diversity for all populations sampled in this study. We calculated *H* for each population; populations with the highest *H* values (*H* >10) would be considered as priority units for protection.

## Results

### cpDNA sequences and haplotypes of *B*. *sinensis*

Across the 23 sampled populations of *B*. *sinensis*, four nucleotide substitutions and one 6-bp indel were observed in *rps*8-*rps*11, which was 832 bp in length after alignment. As to *rps*16-*trn*Q, one homopolymer repeat polymorphic site and two nucleotide variants were detected over the aligned length of 670 bp. Within *trn*T-*trn*L region, there were three substitutions, two indels, and two cpSSRs sites over an aligned length of 761 bp. The number of haplotypes recognized in *rps*8-*rps*11, *rps*16-*trn*Q and *trn*T-*trn*L were five, three, and ten respectively. In total, 13 haplotypes were defined by combining the sequences of the three regions.

### Genetic diversity and spatial patterns of cpDNA variation

The haplotype diversity (*h*) of all populations, excluding sites with gaps, was 0.645. We found a very limited number of haplotypes within each population, with only a single haplotype found in 19 of the 23 populations (Table 1). Among these 19 populations, 11 had haplotype A. Only four populations possessed two or three haplotypes. The highest haplotype diversity, 0.682, was found in the HZ population from Guangdong province, which contained a large number of adult individuals. The highest nucleotide diversity (π = 0.182 × 10^−3^) was found in the LS population of Guangxi province, followed by the HZ population. Total genetic diversity (*h*_T_ = 0.739, *V*_T_ = 0.742) was considerably higher than the average genetic diversity at the populational level.

According to our AMOVA result, the majority of variation existed among populations (98.09%, *P* < 0.001), and only 1.91% was distributed within populations. *N*_ST_ was significantly higher than *G*_ST_ (*N*_ST_ = 0.973, *G*_ST_ = 0.887, *P* < 0.0001), which indicated a phylogeographic structure of the haplotype distribution in *B*. *sinensis*. SAMOVA produced various sets of boundaries depending on the number of groups (*K*), which we arbitrarily set before analysis (Table 3). The proportion of genetic variation among groups (*F*_CT_) was highest and the proportion of genetic variation between populations within groups (*F*_SC_) was lowest when *K* = 5, which indicated that the variation in cpDNA in *B*. *sinensis* could be divided into five geographical groups.

**Table 3:** Comparison of group composition and fixation indices for groupings of the 23 Chinese *Bretschneidera sinensis* populations detected by SAMOVA.

No. groups (k)	Group composition	*F*_SC_	*F*_CT_	*F*_ST_
2	Group I: CY JN LQ SX LTJ GD NP GP TB LN XHS LZ MS Group II: LP DA LS PB QY CH HZ JX XN ES	0.93058	0.83573	0.98860
3	Group I: CY JN LQ SX LTJ GD NP GP TB LN XHS LZ MS Group II: LP DA LS PB CH HZ JX XN ES Group III: QY	0.89807	0.88627	0.98841
4	Group I: CY JN LQ SX LTJ GD NP GP TB LN XHS LZ MS Group II: LP DA LS PB JX XN ES Group III: QY Group III: CH HZ	0.83992	0.92364	0.98778
5	Group I: CY JN LQ SX LTJ GD NP GP TB LN XHS LZ MS Group II: QY Group III: LP DA XN ES Group IV: CH HZ Group V: LS PB JX	0.67089	0.96096	0.98715
6	Group I: the same as k = 5 Group II: LP DA XN ES Group III: QY Group IV: LS PB JX Group V: CH HZ Group VI: MS	0.67963	0.95810	0.98658
7	Group I: the same as k = 5 Group II: CH HZ Group III: LP DA XN Group IV: LS PB JX Group V: ES Group VI: QY Group VII: MS	0.10182	0.98494	0.98648

### Geographic distribution of cpDNA haplotypes

The geographic distribution of the 13 haplotypes identified in the 23 populations is shown in Fig 1A. Haplotype A was the most common and widespread; 12 populations were observed in the eastern and central parts of the species’ range, including Zhejiang, Fujian, Jiangxi, and Guangdong provinces. There were nine unique haplotypes that each occurred in only one population. Haplotype D existed in four populations, and haplotypes E and H were each found in two populations (Fig 1A).

### Phylogenetic relationships among haplotypes of *B*. *sinensis*

The relationships between the 13 haplotypes defined by all mutations were revealed in the networks constructed by NETWORK program (Fig 1B), Haplotypes D, E, and F occupied the most interior positions in the two respective networks. Haplotypes C, D, E, F occurred in a similar geographic range consisting of LP, XN, DA, LS, and PB populations. The network also revealed that F and K were the most differentiated, separated from one another by four mutations (one indel with 6 bp, two nucleotide substitutions, and one cpSSRs site).

**Fig 2:**
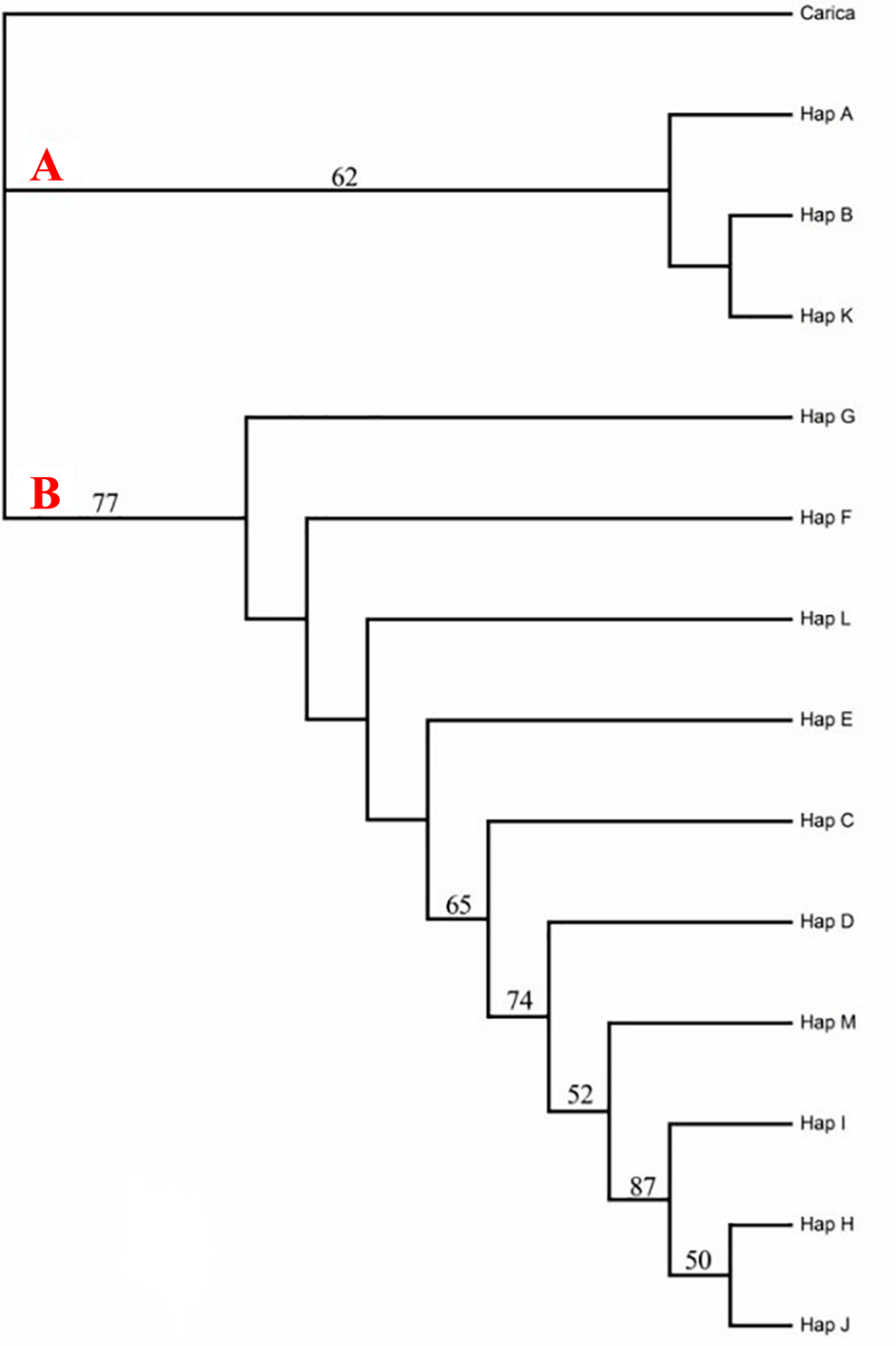
Neighbor-joining tree for haplotypes of *Bretschneidera sinensis* in China. Neighbor-joining tree of 13 haplotypes (including cpDNA SSRs) of *B*. *sinensis*. Note: Red letters A and B stand for different clades. Bootstrap values (higher than 50) are given above the branches.

The network was largely consistent with the neighbor-joining (NJ) tree topology, in which two major lineages can be identified (Fig 2). Lineage A consisted of populations located in the Mt. Wuyi, Mt. Nanling and Taiwan; lineage B comprised populations from the rest of the species’ range, including populations in Guangdong, Guangxi, Guizhou, Hubei, Hunan and Yunnan provinces.

### Historical demographic analysis

Both Tajima’s *D* and Fu and Li’s D* and F* neutrality tests exhibited non-significant positive values at the specific level (Table 4), indicating that the species did not appear to have undergone range expansion. However, when geographic groups were tested separately, significant positive values were found for both Tajima’s *D* and Fu and Li’s F* values in the central part of China, which was evident that populations of *B*. *sinensis* in this region had experienced shrinkage.

**Table 4:** Neutral tests of all *Bretschneidera sinensis* populations and geographic regions.

Population	*D*	D*	F*
Total	1.29224	1.25354	1.51921
Group I	—	—	—
Group II	—	—	—
Group III	-0.10401	-0.88132	-0.75733
Group IV	-0.13252	0.64197	0.49903
Group V	-0.44826	0.58708	0.34490
SW China	1.53133	0.64952	1.01002
Central China	2.79455**	1.06603	1.90238*
Eastern China	——	——	——
Lingnan	1.75657	1.25580	1.69170
Southern China of mt. Nanling	1.64317	1.25515	1.64539
Northern China of mt. Nanling	2.23250*	1.00321	1.667435
LP	-1.14053	-1.32974	-1.44334
LS	0.05455	0.75202	0.78728
HZ	0.05455	0.75202	0.78728

Note

*: P<0.05

**: P<0.001

Groups I-V correspond to their counterpart in Table 3 (k = 5); the compositions of each geological group are listed below.

SW China: LP, PB

Central China: CY, DA, GD, LN, XN, ES

Eastern China: JN, LQ, GP, NP, TB

Lingnan: SX, LTJ, LS, QY, CH, HZ, JX, XHS, LZ, MS

Southern China of mt. Nanling: SX, LTJ, LS, PB, QY, CH, HZ, JX, XHS, LZ

Northern China of mt. Nanling: CY, JN, LQ, LP, GD, DA, NP, GP, TB, LN, XN, ES, MS

**Fig 3:**
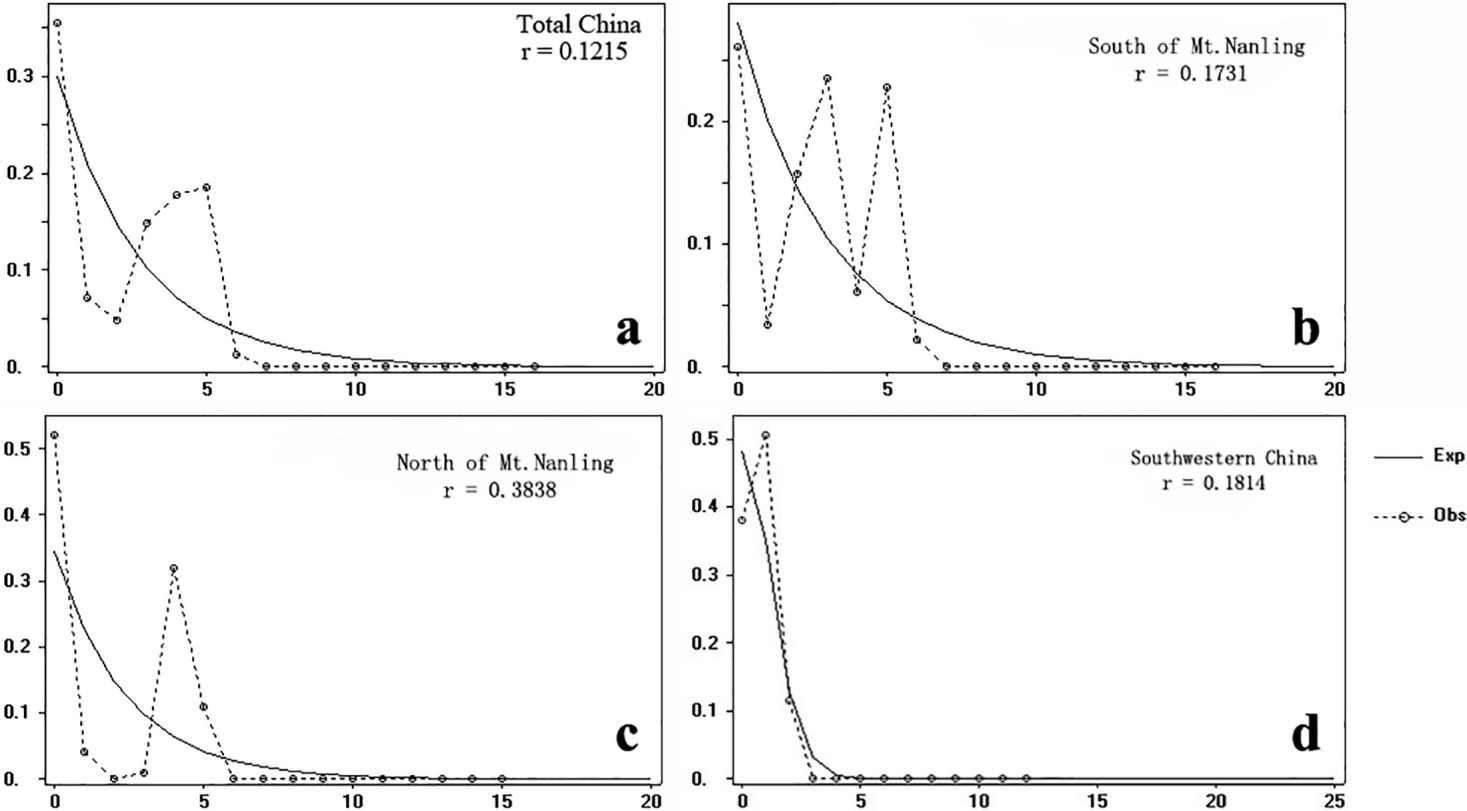
Mismatch distribution analysis of *Bretschneidera sinensis* among different biogeogrphical groups. (A) Total China. (B) South of Mt. Nanling. (C) North of Mt. Nanling. (D) Southwestern China.

Mismatch distribution analysis with DnaSP showed multi-modal waves at the specific level (Fig 3), which indicated that *B*. *sinensis* had not gone through apparent range expansion. When geographic groups were tested separately, mismatch distribution of the populations in southwestern China produced a unimodal value (Fig 3D), which showed that populations in this region experienced range expansion.

### Priority protected unit analysis

Three ancestral haplotypes (D, E, and F) and 10 derived haplotypes (A, B, C, G, H, I, J, K, L, and M) were detected in this study (Fig 1B). Within these haplotypes, there were nine private haplotypes (B, C, F, G, I, J, K, L, and M), four common haplotypes (A, D, E, and H), and four rare haplotypes (B, C, I, and J). Seven populations, LP, LS, HZ, CY, DA, PB and XN had *H* values >10 (35.7, 25.8, 23.7, 19, 12, 12 and 12, respectively; see Table 5). We proposed that these seven populations could be designated as the priority protected units of *B*. *Sinensis*.

**Table 5:** *H* values for *Bretschneidera sinensis*.

Population	Haplotype	*Ha*	*Hd*	*He*	*Hr*	*Hc*	10*πi/π*	*H*
1. ES	M	0	3	5	0	0	0	8
2. GD	A	0	3	0	0	3	0	6
3. DA	D	7	0	0	5	0	0	12
4. XN	D	7	0	0	5	0	0	12
5. JN	A	0	3	0	0	3	0	6
6. LQ	A	0	3	0	0	3	0	6
7. CY	A, B	0	3+3	5	5	3	0	19
8. LN	A	0	3	0	0	3	0	6
9. LP	D, E, C	7+7	3	5	5+5	3	0.7	35.7
10. NP	A	0	3	0	0	3	0	6
11. GP	A	0	3	0	0	3	0	6
12. PB	F	7	0	5	0	0	0	12
13. LS	D, E	7+7	0	0	5+5	0	1.8	25.8
14. JX	L	0	3	5	0	0	0	8
15. CH	H	0	3	0	5	0	0	8
16. HZ	H, I, J	0	3+3+3	5+5	0	3	1.7	23.7
17. SX	A	0	3	0	0	3	0	6
18. QY	G	0	3	5	0	0	0	8
19. LZ	A	0	3	0	0	3	0	6
20. LTJ	A	0	3	0	0	3	0	6
21. XHS	A	0	3	0	0	3	0	6
22. MS	A	0	3	0	0	3	0	6
23. TB	K	0	3	5	0	0	0	8

## Discussion

### Genetic diversity and population differentiation in *B*. *sinensis*

Comparatively high total haplotype diversity (*h*_T_ = 0.739) was demonstrated among the 23 populations of *Bretschneidera sinensis* in this study. High cpDNA diversity has been reported in other long-lived endemic or relict species, such as *Eurycorymbus cavaleriei* [19] (*h*_T_ = 0.834) and *Taxus wallichiana* [49] (*h*_T_ = 0.884). There are two possible explanations for this phenomenon. On one hand, relict plant species with a long evolutionary history may have accumulated a large amount of genetic variation [50, 51]. On the other hand, the wide geographic range of *B*. *sinensis* may represent a large effective population size from which significant polymorphism could have arisen.

Haplotype diversity within populations was quite low (*h*_S_ = 0.083). In contrast, high diversity of *B*. *sinensis* was characterized by remarkable differentiation among populations (*G*_ST_ = 0.887). High *G*_ST_ is common in the cpDNA of angiosperm tree species [52], which always lead to a high total haplotype diversity (*h*_T_). While compared with the mean overall *G*_ST_ value for angiosperms (*G*_ST_ = 0.637) [27], genetic differentiation in *B*. *sinensis* is considerably greater. A similar result was produced by AMOVA analysis, which showed that almost all of the variation existed among, rather than within, populations. Low genetic diversity within populations, combined with strong population differentiation, revealed that the *B*. *sinensis* populations were highly isolated from each other. Severe bottlenecks and/or genetic drift associated with small effective population sizes for maternally inherited markers have usually been responsible for such phenomena [30]. The 23 natural populations investigated in our field survey occurred in isolated and fragmented locations. Range fragmentation may have reduced effective population size and gene flow, and consequently led to low genetic diversity within populations and strong genetic differentiation across the species. Population differentiation resulted from urbanization-caused range fragmentation has been documented for several other relict species with a geographic range similar to *B*. *sinensis* [19, 53, 54,].

Short-distance dispersal in *B*. *sinensis* seeds may have further contributed to the high population differentiation observed in this species. The seeds of *B*. *sinensis* are large (mean diameter: 1.41–1.52 cm) [55] and their arils are smooth, rendering them unlikely to be dispersed over long distances by wind or animals. We did not observe animals feeding on the *B*. *sinensis* seeds; it is likely that its seeds are spread by gravity. Population differentiation of the chloroplast genome was found to be strongly influenced by the mode of seed dispersal in different ways, and species with seeds dispersed by gravity showed higher genetic differentiation than those with wind-dispersed seeds [26], consistent with the strong genetic differentiation observed in *B*. *sinensis*.

### Refugia, demographic history and phylogeographic implications

There are several criteria for the recognition of glacial refugia: they must have harbored both ancestral and endemic haplotypes [56]; they must possess high levels of genetic diversity; and they must have had comparatively stable ecological conditions during periods of environmental fluctuation, which made the accumulation of genetic diversity possible [29, 57]. Coalescent theory maintains that older alleles occupy interior nodes of a haplotype network [58]. In our network of 13 haplotypes, D, E, and F were the most interior and can be considered as ancestral (Fig 1B). However, owing to an incomplete sampling in Yunnan province, it is also possible to regard the present putative “ancestral haplotypes” as descendants of the “true ancient one”. To solve such a problem, a better sampling for *B*. *sinensis* populations, especially in Yunnan province, is promising and planned for our next field trip.

Based on these criteria, the East Yungui Plateau in southwestern China, where populations LP and LS are located, could be regarded as a glacial refuge of *B*. *sinensis*. Populations in this area possess high levels of both nucleotide and haplotype diversity. In addition, all of the haplotypes found in this area (C, D, and E, of which D and E are ancestral) are endemic in the East Yungui Plateau (Fig 1B), which is located in the southeast of Qinghai-Tibet Plateau, south of Qinling Mountains, suggesting that the East Yungui Plateau may have been less influenced by the Neogene and Quaternary glaciations than other geographic areas in China. The Yungui Plateau and its adjacent regions have long been considered an important center of origin for other members of the East Asiatic flora [59]. Many fossils of relict plants are found in this area, explicating it as one of the world’s biodiversity hotpots [60], and thus a glacial refugium of many angiosperms and gymnosperms in China [61]. These species include *Eurycorymbus cavaleriei* [19], *Ginkgo biloba* [29], and *Cunninghamia konishii* [62], etc. The East Yungui Plateau is also a refugium for several taxa of Sapindaceae, a family that is closely related to Akaniaceae, as represented by *Handeliodendron bodinieri* and *Eurycorymbus cavaleriei* [19].

Furthermore, we discovered that the southeastern part of Yunnan Province and its adjacent regions, where the PB population is found, is another glacial refugium of *B*. *sinensis* and represents another biodiversity hotspot [60]. Although we did not detect particularly high nucleotide or haplotype diversity in this area, haplotype F, one of the most ancestral, occurred here. This area, with its historically temperate and humid climate, which is conducive to the preservation of ancient plants, may be another center of relict species [63]. Whereas PB is the only population being collected in Yunnan, a scenario of multi-refugia in this province may be implied. For example, Hengduan Mtn., which lies in SW Yunnan and abuts Tibet, is another potential refugium candidate according to some old specimen labels.

The Nanling Mountains and adjacent regions, where six populations of *B*. *sinensis* are located, typified the third hotspot of biodiversity in China, even though there are no ancestral haplotypes in this area. This area does, however, harbor high levels of nucleotide and haplotype diversity. Four haplotypes (G, H, I, J) occur in this area and all are endemic. One possible explanation the high genetic diversity observed in this region may have resulted from genetic admixture after dispersal from adjacent regions.

**Fig 4:**
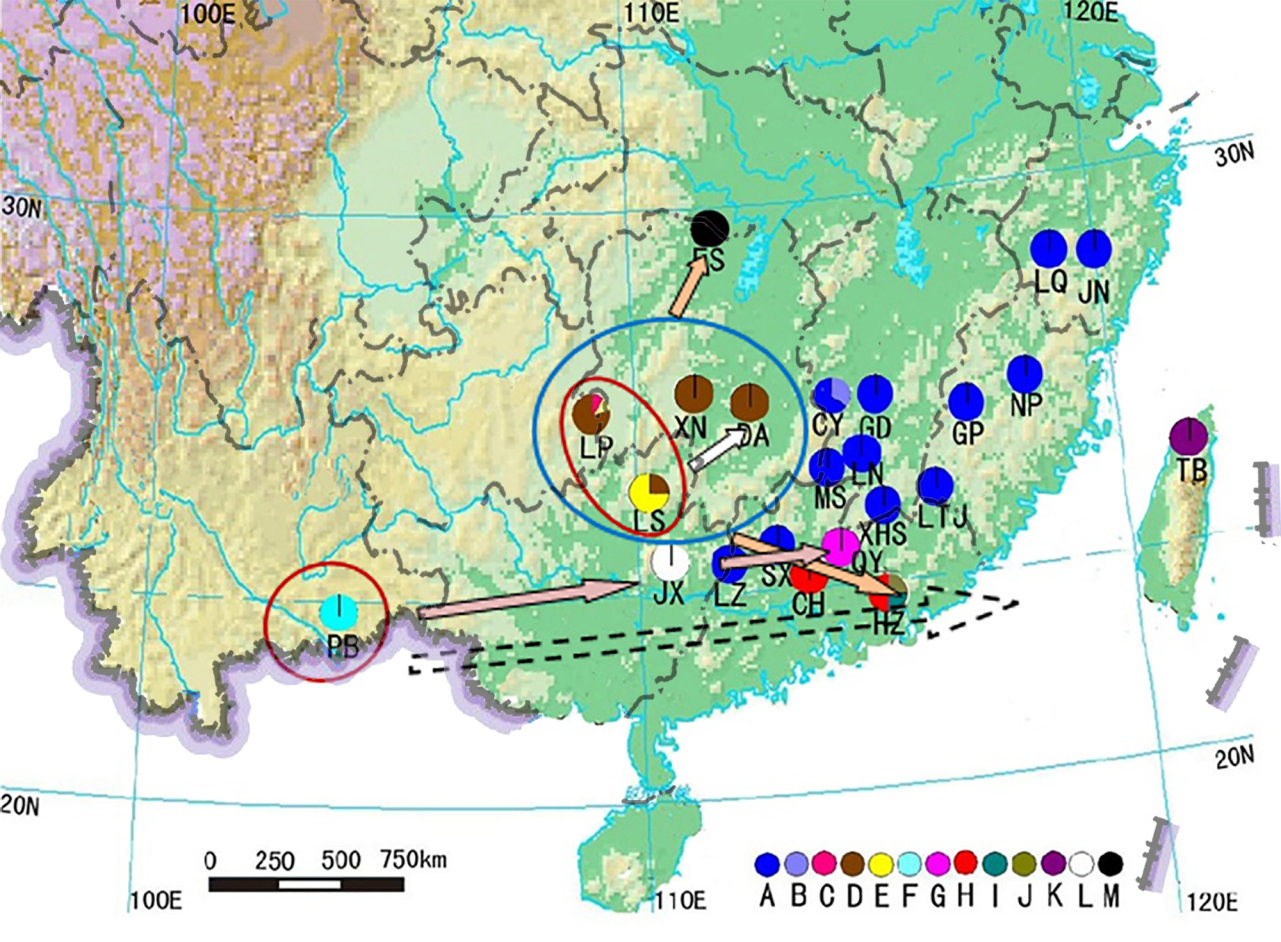
Possible migration and expansion routes of *Bretschneidera sinensis* in China. Letters below the colorful circles in the map stand for the population names; different colorful circles are to distinguish the haplotypes (see Legend); Three larger circles stands for refugia; arrows indicate possible migration directions.

According to the abovementioned inference, *Bretschneidera sinensis* may have the following history of population dynamics (see Fig 4). The East Yungui Plateau refugium may be the source of populations from southeastern Hunan and Hubei provinces. After last glacial period in Quaternary, evergreen broadleaf forest in South China reached its flourish. Mismatch distribution analysis in southwestern China shows that populations in the East Yungui Plateau refugium had undergone range expansion under the circumstances. Contrarily, populations from the Mt. Wuyi, Mt. Nanling and mountains in Taiwan may be derived from the other refugium, southeastern Yunnan, after its initial eastern colonization of *B*. *sinensis*.

Haplotype A, which is not an ancient haplotype, occurs throughout most populations from the Mt. Nanling and Mt. Wuyi, providing strong support for a postglacial range expansion from a single refugium to its present range. The network of haplotypes shows that haplotype A is closely related to haplotype K, which is closely related to the ancestral haplotype F found in eastern Yunnan Province. Thus, eastern Yunnan is likely a source for the colonization of *B*. *sinensis* into eastern China. Postglacial dispersal and range expansion also occurred in other relict plants in China, such as *Cercidiphyllum* [64], *Davidia* [65, 66], *Euptelea* [67] and *Tetracentron* [68].

### Conservation implications for *Bretschneidera sinensis*

Seven *Bretschneidera sinensis* populations: CY, DA, HZ, LP, LS, PB and XN had the highest *H* values (*H* >10). Thus, we recommend these populations to be the priority conservation units for this species. These seven populations exhibit the following characteristics. First, some (DA, LP, LS, PB and XN) are located in the inferred refugium areas, which retain ancestral haplotypes of the species. Second, some of these populations (PB, LP, and CY) possess private or rare haplotypes. If these populations become extinct, the diversity of *B*. *sinensis* would be diminished. Thirdly, several populations (HZ and CY) exhibit high genetic diversity. Protection of these populations could best preserve the diversity of this species to the maximum extent possible. Fortunately, most populations of *B*. *sinensis* are located in national nature reserves, including these seven priority populations. Finally, the seven priority populations can be used for *ex situ* conservation. Given the high degree of among-population differences in genotype, it is likely that genetic diversity will increase substantially by the exchange of genetic materials that takes place among those populations.
